# Engineered bacteria reverses tumor cuproptosis resistance via cancer-associated fibroblast reprogramming for enhanced immunotherapy

**DOI:** 10.1016/j.mtbio.2026.103603

**Published:** 2026-08-26

**Authors:** Yuhao Chen, Xiaorui Geng, Ning Su, Phei Er Saw, Yun Li, Bin Liu, Yingjia Li, Zhen Yuan

**Affiliations:** a Department of Public Health and Medicinal Administration, Faculty of Medicine, University of Macau, China; b Centre for Cognitive and Brain Sciences, University of Macau, China; c Department of Medicine Ultrasonics, Nanfang Hospital, Southern Medical University, Guangzhou, China; d Medical Research Center, Sun Yat-sen Memorial Hospital, Sun Yat-sen University, Guangzhou, China; e Guangdong Yunzhao Medical Technology Co., LTD, Guangzhou, China; f Department of Emergency, Zhujiang Hospital, Southern Medical University, Guangzhou, Guangdong Province, China

**Keywords:** Cuproptosis, Immunotherapy, Cancer-associated fibroblasts (CAFs), Bacteria, Tumor microenvironment

## Abstract

Cuproptosis, a novel modality of mitochondrial copper-induced cell death, holds great therapeutic potential against cancer. However, its efficacy is limited by cancer-associated fibroblasts (CAFs), which confer the cuproptosis resistance in tumor by upregulating the copper exporter ATP7A. In this study, we developed an engineered bacteria system, PCuS-MG, for synergistic cuproptosis and immunotherapy via CAFs reprogramming. PCuS-MG consists of *E. col*i MG1655 modified with pirfenidone-loaded copper sulfide nanoparticles. Upon intravenous administration and 1064 nm laser irradiation, PCuS-MG accumulates in tumors and releases Cu^2+^ and pirfenidone. The released Cu^2+^ synergistically triggers cuproptosis. Meanwhile, pirfenidone reprograms CAFs via TGF-β signaling inhibition and glucose metabolism modulation, thereby downregulating tumor ATP7A to reduce cuproptosis resistance. In a triple-negative breast cancer model, PCuS-MG potently inhibited primary and distant tumor growth and suppressed lung metastasis by remodeling the immune microenvironment. This study reveals the pivotal role of CAFs in cuproptosis resistance and introduces PCuS-MG as a novel platform that synergizes metal ion-induced death with CAFs reprogramming, offering a promising strategy for tumor therapy.

## Graphical abstract

**Figure:**
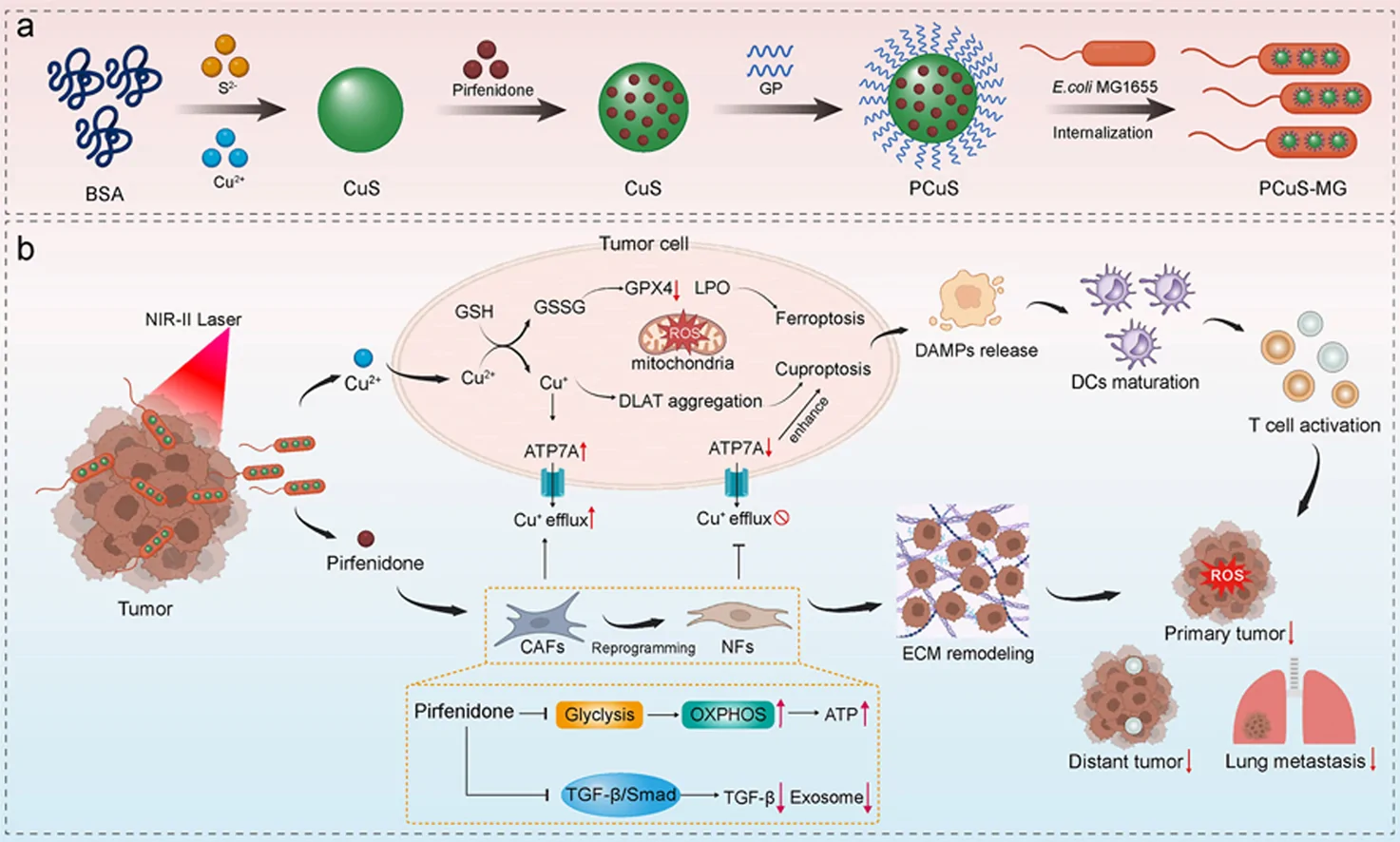

## 1 Introduction

Tumors pose a significant threat to human health, with major challenges including drug resistance and high metastasis rates [1]. In recent years, the rapid advancement of metal ion-dependent cell death therapies—including cuproptosis and ferroptosis—has offered new hope for tumor treatment [[2], [3], [4], [5]]. However, the robust adaptive regulatory mechanisms of tumors induce resistance to cuproptosis, which limits its overall therapeutic efficacy [6,7]. For instance, ATPase copper transporting alpha (ATP7A) is a key channel protein located on the cell membrane that mediates copper ion transport [8]. In various types tumor cells (e.g., triple-negative breast cancer cells), ATP7A expression is significantly upregulated, accelerating intracellular copper efflux. By reducing intracellular copper concentrations, tumor cells acquire resistance to copper-induced apoptosis [[9], [10], [11]]. The combination of cuproptosis and immunotherapy has been demonstrated in numerous studies to yield superior therapeutic outcomes. Cuproptosis can induce immunogenic cell death (ICD) and promote the release of damage-associated molecular patterns (DAMPs), thereby activating anti-tumor immune responses [12]. However, the benefits of this combination therapy are not universally observed across all patients, highlighting the necessity of investigating cuproptosis resistance mechanisms to optimize the efficacy of cuproptosis-immunotherapy [13,14].

Cancer-associated fibroblasts (CAFs) are the most abundant stromal cells in the tumor microenvironment (TME), creating a favorable niche for tumor growth and metastasis through interactions with tumor cells and autocrine/paracrine signaling [15,16]. Notably, CAFs exhibit intrinsic resistance to cuproptosis and can activate angiogenesis by secreting vascular endothelial growth factor (VEGF), thereby reducing copper ion concentrations within the TME [17,18]. Activated CAFs in the TME undergo specific changes in their secretory profile, including the secretion of exosomes containing miR-148b-3p and upregulation of TGF-β1, which are associated with enhanced ATP7A expression, cuproptosis resistance, and invasiveness in tumor cells [19]. Beyond potentiating cuproptosis resistance in tumor cells, CAFs also modulate the tumor immune microenvironment to induce immune escape [20]. For instance, CAFs produce large amounts of collagen and matrix metalloproteinases (MMPs), leading to extracellular matrix (ECM) stiffening that impedes immune cell infiltration and forms an immunosuppressive microenvironment [21,22]. However, most current studies still focus on tumor cells themselves rather than the potential critical role of CAFs in mediating cuproptosis resistance. Therefore, elucidating the CAF-sustained mechanisms of cuproptosis resistance is crucial for developing strategies to overcome cuproptosis-immunotherapy resistance.

Recently, anti-CAFs agents, including pirfenidone and navitoclax, exhibit potent anti-tumor efficacy. However, their clinical application is limited by uncontrollable toxicity and systemic adverse effects. For example, pirfenidone can effectively inhibit the activation of CAFs and promote the remodeling of the abnormal ECM by suppressing the TGF-β/SMAD signaling pathway. Furthermore, pirfenidone may also alter the metabolic pattern of CAFs, thereby achieving the reprogramming of CAFs via multiple mechanisms [23,24]. Nevertheless, pirfenidone treatment may cause gastrointestinal discomfort and potential hepatotoxicity in patients. Encapsulating anti-CAFs agents into nanoparticles holds promse for addressing these issues. Copper-based nanoparticles not only optimize the *in vivo* circulation and bioavailability of drugs but also release copper ions to induce cuproptosis, ultimately enhancing the overall therapeutic efficacy [25]. Furthermore, constructing a "Trojan bacterium" system by combining drug-loaded copper-based nanoparticles with bacteria can leverage the inherent hypoxia tropism of bacteria to achieve highly selective targeted drug delivery to the tumor microenvironment [26,27]. Therefore, developing a multifunctional therapeutic platform capable of simultaneously modulating CAFs and eliminating tumor cells is expected to provide a feasible strategy for remodeling the tumor stroma and reducing treatment resistance during cuproptosis-immunotherapy.

**Scheme 1:**
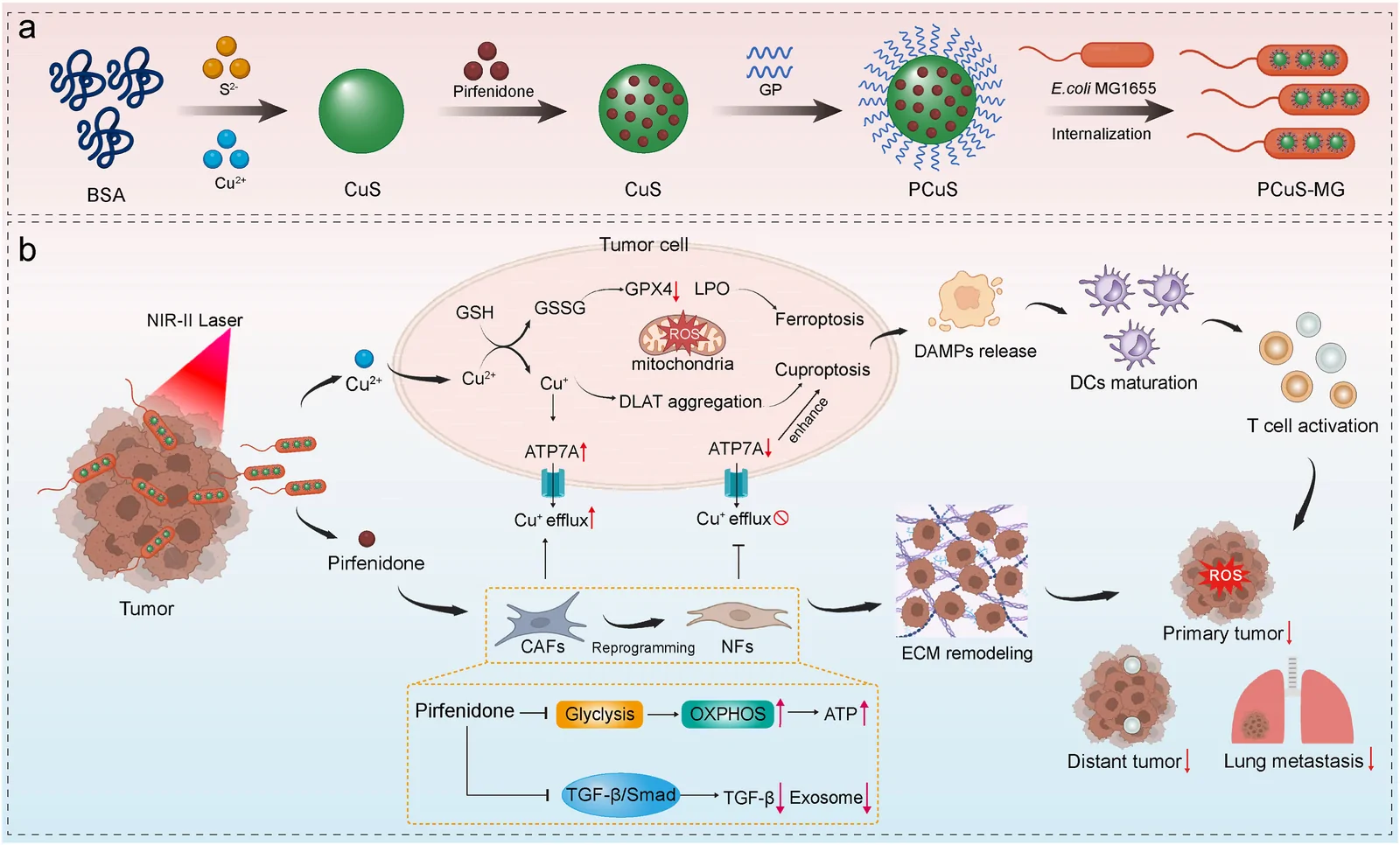
Schematic of the engineered bacteria (PCuS-MG) system for enhanced cuproptosis and immunotherapy. Following targeted delivery to the tumor, PCuS-MG releases Cu^2+^ and pirfenidone under 1064 nm laser irradiation. The elevated intracellular Cu^2+^ levels induce concurrent cuproptosis and ferroptosis in tumor cells, triggering ICD and the release of DAMPs. Simultaneously, the released pirfenidone inhibits CAFs, which suppresses the upregulation of the copper exporter ATP7A in tumor cells. The combination of robust ICD and ECM remodeling promotes DCs maturation and cytotoxic T cell activation, culminating in a potent anti-tumor immune response.

In this study, we developed an engineered bacteria therapeutic platform (PCuS-MG), comprising pirfenidone-encapsulated copper sulfide nanoparticles (PCuS) and *Escherichia coli* (*E. coli*) MG1655. This platform enables precise targeting of the TME to inhibit CAF activation and enhance the efficacy of cuproptosis-immunotherapy against tumors (Scheme 1a). Following intravenous injection, PCuS-MG exhibited efficient tumor accumulation. Upon irradiation with a 1064 nm near-infrared (NIR) laser, this system triggered the controlled release of pirfenidone and copper ions (Cu^2+^), which could significantly induce cuproptosis in tumor cells. Furthermore, pirfenidone markedly promoted the reprogramming of CAFs into a quiescent state by simultaneously regulating the TGF-β/SMAD signaling pathway and glucose metabolism. Moreover, it alleviated tumor cell resistance to cuproptosis-immunotherapy through multiple mechanisms, including downregulating the overexpression of the copper efflux transporter ATP7A in tumor cells and remodeling the dense extracellular matrix (ECM). Notably, PCuS-MG not only promoted dendritic cells (DCs) maturation but also induced M1 macrophage polarization and enhanced CD8^+^ T cell infiltration (Scheme 1b). Experiments in a mouse model of triple-negative breast cancer (TNBC) demonstrated that PCuS-MG significantly boosted the efficacy of cuproptosis-immunotherapy and effectively inhibited distant tumor invasion and lung metastasis. Our study indicates the role of CAFs in mediating tumor cuproptosis resistance and achieves potent anti-tumor therapy through the development of an engineered bacteria therapeutic platform.

## 2 Results

### 2.1 Evaluation of the cuproptosis resistance in tumor cells mechanism mediated by CAFs

**Fig. 1:**
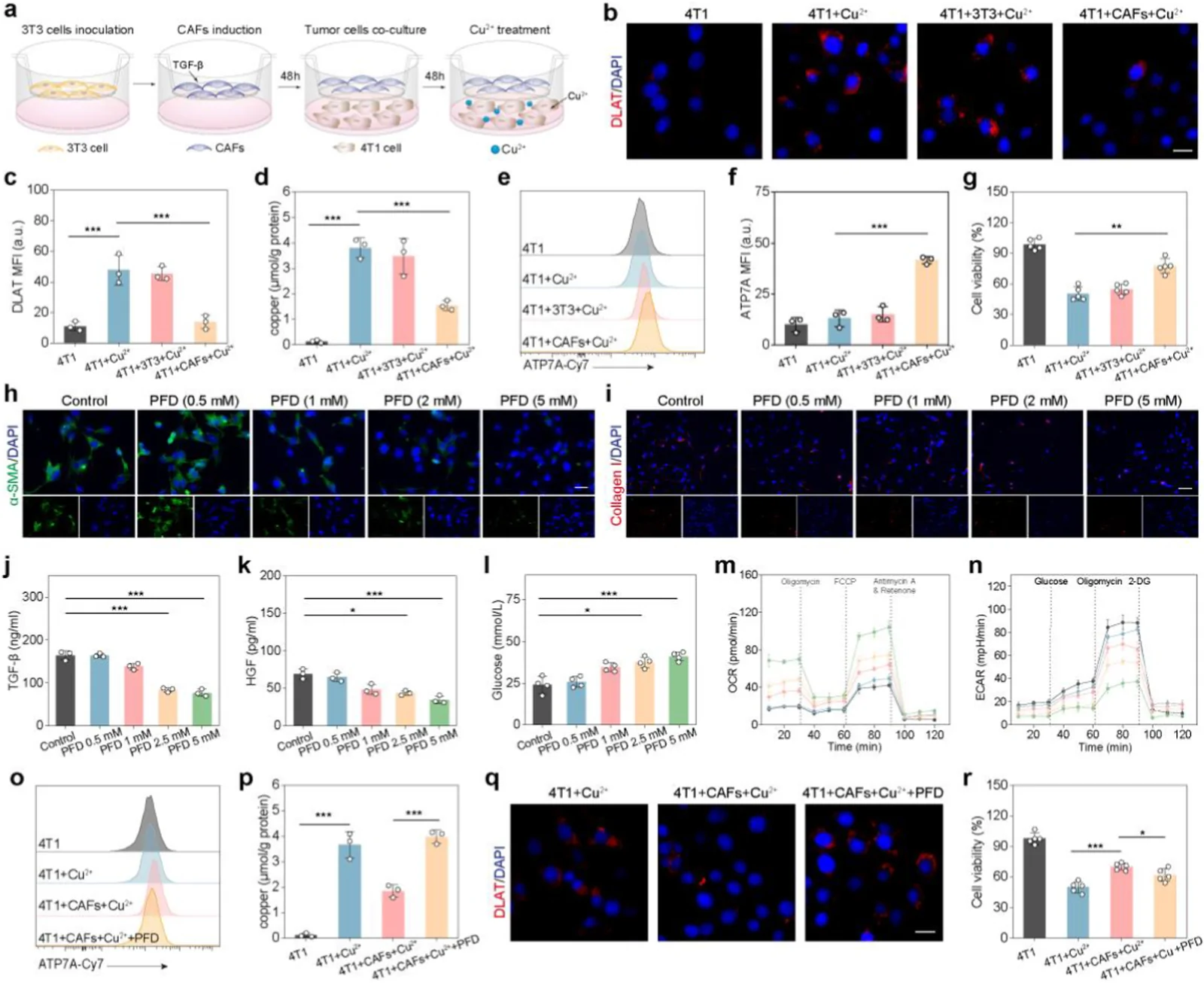
**CAFs induce tumor cell resistance to cuproptosis, and PFD reverses the resistance by reprogramming CAFs.** (a) Schematic illustration of the experimental procedure. 3T3 fibroblasts were induced into CAFs by TGF-β stimulation, followed by co-culture with 4T1 cells, and subsequent Cu^2+^ treatment to assess cuproptosis resistance. (b-c) Immunofluorescence staining of DLAT in 4T1 cells under different treatment. Scale bar = 25 μm. (d) Intracellular copper ion levels in 4T1 cells under the indicated treatments. (e-f) Flow cytometry of ATP7A expression in 4T1 cells under different conditions. (g) Cell viability of 4T1 cells under the indicated treatments. (h, i) Immunofluorescence staining of α-SMA and collagen I in CAFs treated with different concentrations of PFD. Scale bar = 50 μm. (j, k) TGF-β and HGF secretion in CAFs treated with PFD. (l) Glucose in CAFs treated with PFD. (m) Oxygen consumption rate (OCR) in CAFs treated with PFD. (n) Extracellular acidification rate (ECAR) in CAFs treated with PFD. (o) Flow cytometry of ATP7A expression in 4T1 cells under the indicated treatments. (p) Intracellular copper ion levels in 4T1 cells under the indicated treatments. (q) Immunofluorescence staining of DLAT (red) in 4T1 cells under the indicated treatments. Scale bar = 25 μm. (r) Cell viability of 4T1 cells under the indicated treatments. (For interpretation of the references to colour in this figure legend, the reader is referred to the Web version of this article.)

To dissect the role of cancer-associated fibroblasts (CAFs) in mediating tumor cell resistance to cuproptosis, we established an *in vitro* co-culture model where 4T1 cells were co-cultured with CAFs induced from NIH 3T3 fibroblasts via TGF-β stimulation for Cu^2+^ treatment (Fig. 1a). Immunofluorescence staining for dihydrolipoamide S-acetyltransferase (DLAT), a critical component of the lipoic acid pathway and a marker of cuproptosis, showed that 4T1 cells co-cultured with CAFs (4T1+CAFs + Cu^2+^) displayed markedly weaker DLAT fluorescence intensity compared with 4T1 cells cultured alone (4T1+Cu^2+^) or co-cultured with naive 3T3 fibroblasts (4T1+3T3+Cu^2+^) (Fig. 1b). Quantitative analysis confirmed a significant reduction in DLAT mean fluorescence intensity (MFI) in 4T1+CAFs + Cu^2+^ group (Fig. 1c). Consistent with reduced cuproptosis, intracellular copper ion levels were significantly lower in 4T1+CAFs + Cu^2+^ group than in 4T1+Cu^2+^ group and 4T1+3T3+Cu^2+^ group (Fig. 1d). Flow cytometry analysis revealed that the expression of ATP7A, a copper efflux transporter linked to cuproptosis resistance, was upregulated in 4T1+CAFs + Cu^2+^ group compared with other groups (Fig. 1e–f). CCK-8 assays further demonstrated that 4T1 cell viability in 4T1+CAFs + Cu^2+^ group exhibited significantly higher viability than 4T1+Cu^2+^ group and 4T1+3T3+Cu^2+^ group (Fig. 1g), indicating that CAFs protect tumor cells from Cu^2+^-induced cuproptosis.

To determine whether pirfenidone (PFD), a known inhibitor of CAF activation, could reverse this resistance, we first evaluated its effect on CAF phenotype. Immunofluorescence staining showed that PFD treatment (0.5–5 mM) dose-dependently reduced the expression of α-smooth muscle actin (α-SMA) and collagen I, markers of CAF activation (Fig. 1h–i, Figure S1). ELISA results confirmed that PFD significantly decreased the secretion of transforming growth factor-β (TGF-β) and hepatocyte growth factor (HGF), pro-tumor cytokines produced by activated CAFs (Fig. 1j and k). Metabolic analysis revealed that PFD treatment altered CAF glucose metabolism, as evidenced by increased glucose levels, indicating a suppression of glycolysis (Fig. 1l). Consistent with this, qRT-PCR analysis showed that PFD treatment significantly downregulated the mRNA expression of key glycolytic enzymes, hexokinase 2 (HK2) and lactate dehydrogenase A (LDHA) (Fig. S2). To comprehensively profile this metabolic shift, we also measured the oxygen consumption rate (OCR, an indicator of oxidative phosphorylation) and the extracellular acidification rate (ECAR, an indicator of glycolysis). PFD treatment increased the basal respiratory rate, the maximum respiratory capacity, and ATP production (Fig. 1m, Fig. S3) and exhibited significantly reduced glycolytic rate, maximum glycolytic capacity, and glycolytic reserve (Fig. 1n, Fig. S4). These results indicated that PFD effectively reprograms CAFs from an activated, pro-tumor phenotype to a quiescent state via TGF-β signaling inhibition and metabolism modulation.

Next, we assessed whether PFD could reverse CAF-induced cuproptosis resistance in tumor cells. Flow cytometry analysis showed that PFD treatment (4T1+CAFs + Cu^2+^+PFD) downregulated ATP7A expression in 4T1 cells compared with 4T1+CAFs + Cu^2+^ group (Fig. 1o, Fig. S5). Intracellular copper ion levels were significantly increased in 4T1+CAFs + Cu^2+^+PFD group compared with 4T1+CAFs + Cu^2+^ group (Fig. 1p). Immunofluorescence staining for DLAT showed that PFD restored DLAT expression in 4T1+CAFs + Cu^2+^+PFD group (Fig. 1q). Consistently, cell viability assays demonstrated that 4T1+CAFs + Cu^2+^+PFD group had significantly lower viability than 4T1+CAFs + Cu^2+^ group (Fig. 1r). However, direct PFD treatment to 4T1 cells did not significantly alter ATP7A expression in 4T1 cells (Fig. S6). These results confirm that PFD reverses CAF-induced cuproptosis resistance in tumor cells by downregulating ATP7A and restoring intracellular copper accumulation, thereby sensitizing tumor cells to Cu^2+^-induced cuproptosis.

### 2.2 Preparation and characterization of PCuS-MG

**Fig. 2:**
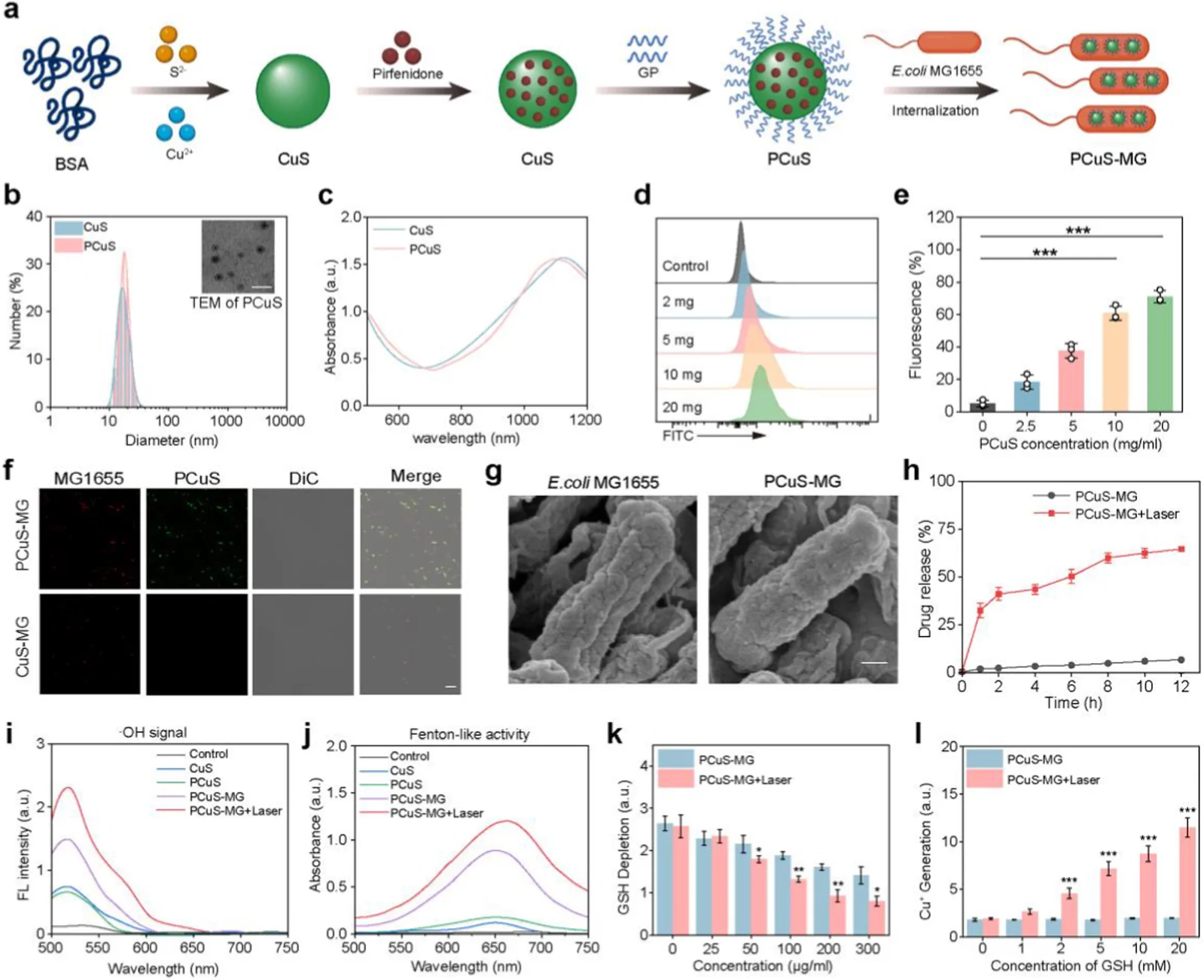
**Characterization of the PCuS-MG.** (a) Schematic illustration of the construction of the PCuS-MG system. (b) Hydrodynamic diameter distribution of CuS and PCuS nanoparticles. (c) UV-Vis-NIR absorption spectra of CuS and PCuS. (d-e) Flow cytometry analysis of PCuS internalization by *E. coli* MG1655 at different concentrations. (f) Fluorescence microscopy images of *E. coli* MG1655 after incubation with FITC-labeled PCuS or CuS. Scale bar = 30 μm. (g) SEM images of native *E. coli* MG1655 and PCuS-MG. Scale bar = 200 nm. (h) *In vitro* drug release profile from PCuS-MG with or without laser irradiation. (i) Hydroxyl radical (·OH) generation detected by HPF probe fluorescence across different treatment groups. (j) Fenton-like catalytic activity assessed by the oxidation of TMB. (k) Glutathione (GSH) depletion capability of different concentrations of PCuS-MG with or without laser irradiation. (l) Generation of Cu^+^ by PCuS-MG following reaction with various concentrations of GSH.

Firstly, we synthesized bovine serum albumin (BSA)-modified copper sulfide nanoparticles (CuS) using a biomineralization approach. The BSA acts as a capping agent, which substantially improves the aqueous solubility and biocompatibility of the nanoparticles. Furthermore, the hydrophobic domains of BSA enable the efficient loading of the hydrophobic drug pirfenidone [28]. To construct the final nanoprobe, glucose polymer (GP) was conjugated to the CuS surface via a Schiff base reaction, resulting in GP-modified, pirfenidone-loaded CuS nanoparticles (designated as PCuS) (Fig. 2a). PCuS nanoparticles revealed a spherical morphology and dynamic light scattering (DLS) measurements indicated an average hydrated particle size of approximately 15.3 nm for PCuS, which was slightly larger than that of the unmodified CuS nanoparticles (∼12.5 nm) (Fig. 2b). Ultraviolet-visible (UV-vis) absorption spectroscopy confirmed that both CuS and PCuS possessed strong absorption in the near-infrared (NIR) region, with a peak at ∼1064 nm (Fig. 2c). The nanoparticles also demonstrated excellent stability, showing no significant size alteration over five days in various media, including PBS buffer, cell culture medium, and medium supplemented with 10% serum (Fig. S6).

Leveraging the known ability of GP-modified nanoparticles to be actively internalized by *Escherichia coli* via ATP-binding cassette (ABC) transporters [29,30], we developed an engineered bacteria system (PCuS-MG) by co-incubating PCuS with *E. coli* MG1655. Flow cytometry analysis confirmed a concentration-dependent uptake of PCuS by the bacteria, which plateaued at a PCuS concentration of 10 mg/mL (Fig. 2d–e). Fluorescence microscopy imaging showed a distinct green fluorescent signal from FITC-labeled PCuS within the PCuS-MG bacteria, whereas no signal was detected when bacteria were incubated with unmodified CuS (Fig. 2f). Scanning electron microscopy (SEM) further supported successful internalization, as the surface morphology of PCuS-MG appeared smooth and was indistinguishable from that of native *E. coli* MG1655 (Fig. 2g). The zeta potential change also confirmed the successful loading into the bacterial vector of PCuS and the encapsulation efficiency of PFD in PCuS-MG was 60.54 ± 4.19% (Fig. S7). We next evaluated the functional capabilities of the PCuS-MG system. Upon irradiation with a 1064 nm laser, the cumulative release of pirfenidone reached 48% within 4 h (Fig. 2h). The viability of *E. coli* MG1655 is known to decrease markedly at irradiation time exceeding 160 s (Fig. S8), suggesting that laser can disrupt the bacterial carrier to trigger drug release while simultaneously inactivating it to mitigate potential toxicity. The oxidative stress-inducing capacity of PCuS-MG was also inspected. Using the hydroxyphenyl fluorescein (HPF) probe, the PCuS-MG + laser group exhibited a significantly higher fluorescence intensity at 515 nm, indicating robust generation of hydroxyl radicals (·OH) (Fig. 2i). This was corroborated by Fenton-like activity assays, which confirmed the strongest catalytic activity in the same treatment group (Fig. 2j). The results illustrated that copper ions released from PCuS-MG can effectively catalyze the production of cytotoxic reactive oxygen species (ROS). Additionally, PCuS-MG consumed glutathione (GSH) in a concentration-dependent manner (Fig. 2k). As glutathione reduces Cu^2+^ to the more toxic Cu^+^, this consumption provides direct evidence supporting an induction of cuproptosis (Fig. 2l).

### 2.3 *In vitro* antitumor effects and mechanisms of PCuS-MG

**Fig. 3:**
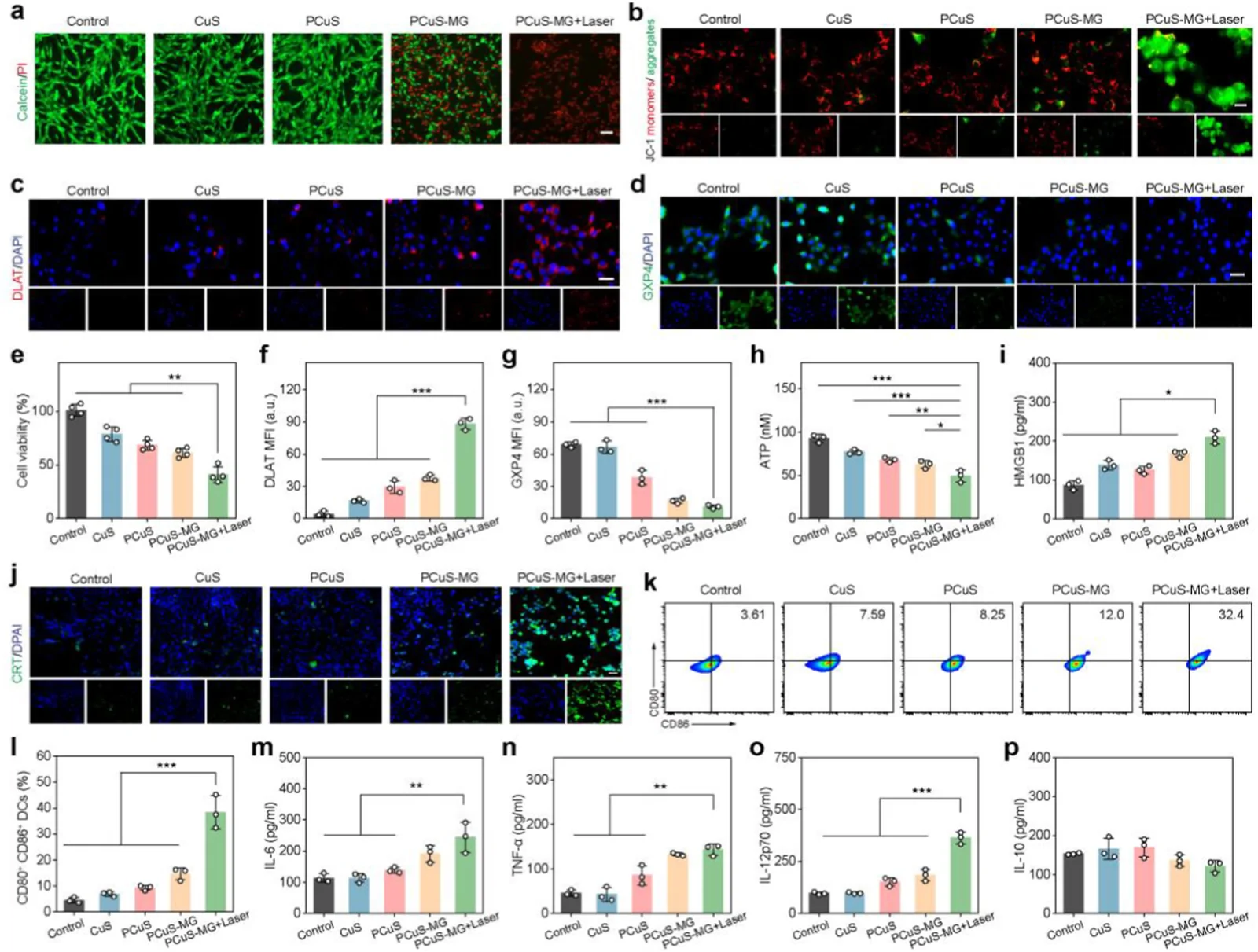
***In vitro* anti-tumor efficacy and immune activation of PCuS-MG.** (a) Live/dead cell staining of 4T1 cells after various treatments. Scale bar = 100 μm. (b) Mitochondrial membrane potential analysis using JC-1 staining in 4T1 cells under the indicated treatments. Scale bar = 20 μm. (c) Immunofluorescence staining of DLAT in 4T1 cells under the indicated treatments. Scale bar = 50 μm. (d) Immunofluorescence staining of GPX4 in 4T1 cells under the indicated treatments. Scale bar = 50 μm. (e) Cell viability of 4T1 cells under the indicated treatments. (f) Quantitative analysis of DLAT mean fluorescence intensity (MFI) in 4T1 cells from (c). (g) Quantitative analysis of GPX4 MFI in 4T1 cells from (d). (h) Intracellular ATP levels in 4T1 cells under the indicated treatments. (i) Extracellular HMGB1 levels released in 4T1 cells under the indicated treatments. (j) Immunofluorescence staining of CRT exposure on 4T1 cells under the indicated treatments. Scale bar = 200 μm. (k-l) flow cytometry of CD80^+^CD86^+^ mature DCs after co-culture with supernatant from differently treated 4T1 cells. (m-p) Pro-inflammatory cytokines (IL-6, TNF-α, IL-12p70) and anti-inflammatory cytokine (IL-10) secretion by DCs after co-culture with supernatant from differently treated 4T1 cells.

To evaluate the therapeutic efficacy and underlying mechanism of PCuS-MG in tumor cells, we then assessed cell viability and cuproptosis induction. First, flow cytometry was used to assess the uptake of PCuS-MG by 4T1 tumor cells. The results demonstrated a time-dependent increase in fluorescence signal within the cells. In addition, the cellular uptake of PCuS-MG was significantly higher than that of PCuS nanoparticles alone, indicating the inherent tumor-targeting ability of the bacteria (Fig. S10). Live/dead staining revealed that PCuS-MG + Laser treatment resulted in a significant increase in dead cells compared to Control, CuS, PCuS, and PCuS-MG groups (Fig. 3a). Consistently, cell viability assays confirmed that PCuS-MG + Laser treatment reduced 4T1 cell viability to 40.89 ± 7.48%, which was significantly lower than all other groups (Fig. 3e). To understand the mechanism of cell death, intracellular ROS levels were measured using the DCFH-DA probe. The PCuS-MG + laser group showed the most intense fluorescent signal, confirming its superior ability to induce ROS accumulation in tumor cells (Fig. S11). Mitochondrial membrane potential analysis using JC-1 staining showed that PCuS-MG + Laser treatment caused a marked reduction in red fluorescence (indicating intact mitochondria) and an increase in green fluorescence (indicating mitochondrial depolarization), suggesting severe mitochondrial dysfunction (Fig. 3b). Immunofluorescence staining for DLAT, a key cuproptosis marker, showed that DLAT expression was significantly upregulated in the PCuS-MG + Laser group (Fig. 3c), with quantitative analysis confirming a ∼3-fold increase in mean fluorescence intensity (MFI) compared to control (Fig. 3f). Conversely, the expression of glutathione peroxidase 4 (GPX4), a critical negative regulator of ferroptosis, was significantly downregulated in the PCuS-MG + Laser group (Fig. 3d), with MFI reduced to ∼20% of control levels (Fig. 3g). This downregulation was accompanied by a significant increase in intracellular lipid peroxidation (LPO), as detected by the C11-BODIPY^581^/^591^ probe, confirming the induction of ferroptosis (Fig. S12). Additionally, intracellular ATP levels were markedly reduced in the PCuS-MG + Laser group (Fig. 3h), consistent with mitochondrial dysfunction. These results demonstrate that PCuS-MG, under NIR laser irradiation, effectively induces cuproptosis and ferroptosis in tumor cells by triggering mitochondrial dysfunction and modulating key cuproptosis-related proteins.

Next, we investigated whether PCuS-MG-induced cell death is immunogenic, a critical factor for activating anti-tumor immunity. We measured the release of high-mobility group box 1 (HMGB1), a DAMP released during ICD. ELISA results showed that HMGB1 levels in the culture supernatant of PCuS-MG + Laser-treated tumor cells were significantly higher than in all other groups (Fig. 3i). Immunofluorescence staining for CRT, another key ICD marker, revealed that PCuS-MG + Laser treatment induced prominent CRT exposure on the tumor cell surface (Fig. 3j), indicating the activation of ICD. To assess the immunostimulatory effects of PCuS-MG-induced ICD, we co-cultured DCs with the supernatant from differently treated tumor cells. Flow cytometry analysis showed that the percentage of CD80^+^CD86^+^ mature DCs was significantly higher in the PCuS-MG + Laser groupcompared to Control, CuS, PCuS, and PCuS-MG groups (Fig. 3k–l). Furthermore, we measured the secretion of key cytokines by DCs. ELISA results showed that PCuS-MG + Laser treatment significantly increased the secretion of pro-inflammatory cytokines, including interleukin-6 (IL-6), tumor necrosis factor-α (TNF-α), and interleukin-12p70 (IL-12p70) (Fig. 3m–o), while the secretion of the anti-inflammatory cytokine interleukin-10 (IL-10) remained unchanged across all groups (Fig. 3p). These results indicate that PCuS-MG-induced ICD effectively promotes DCs maturation and drives a pro-inflammatory immune response, which is essential for initiating anti-tumor immunity.

### 2.4 *In vivo* biodistribution and tumor targeting of PCuS-MG

**Fig. 4:**
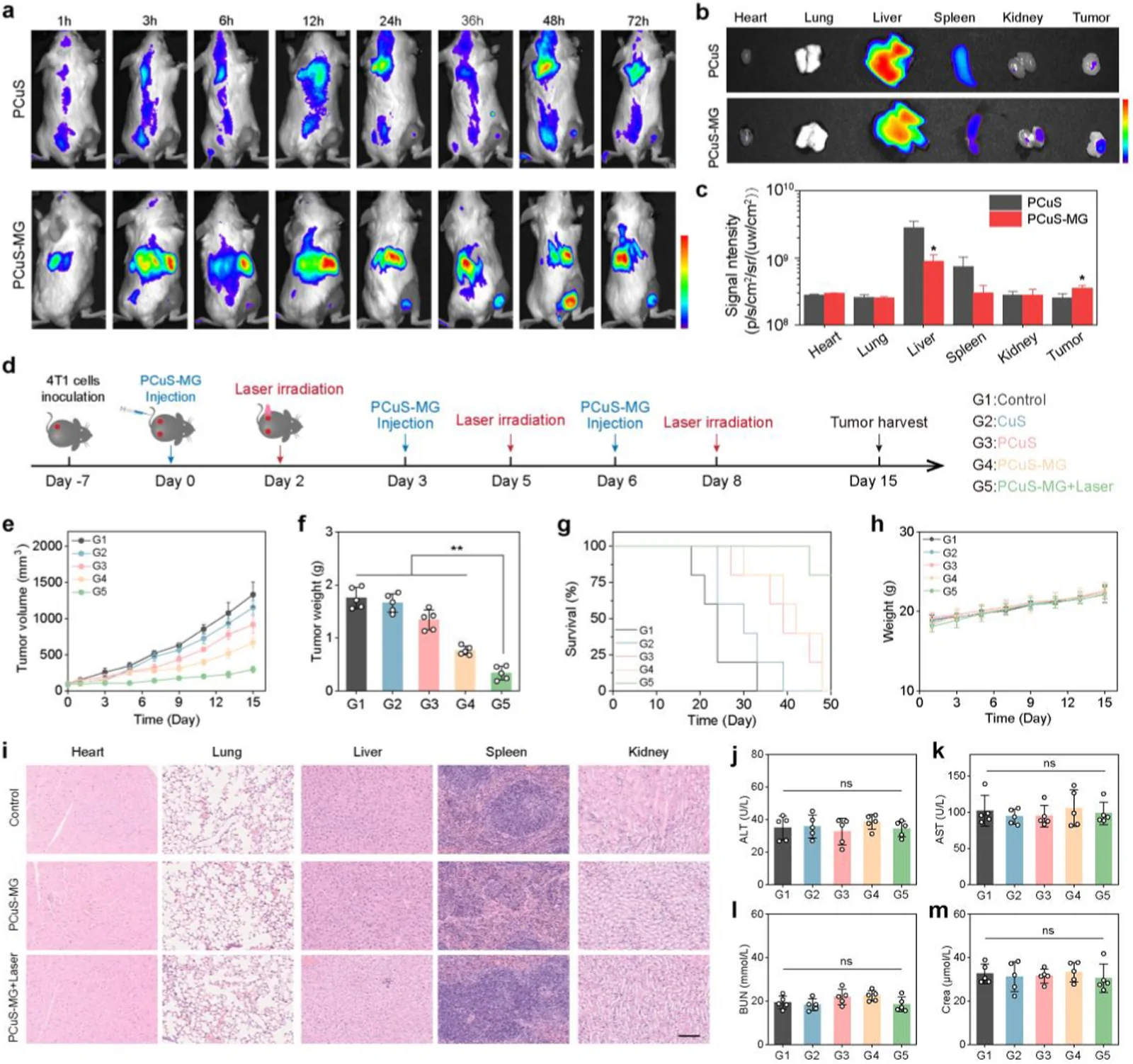
***In vivo* tumor targeting, antitumor efficacy, and biosafety of PCuS-MG in 4T1 tumor-bearing mice.** (a) *In vivo* fluorescence imaging of 4T1 tumor-bearing mice at different time points after intravenous injection of PCuS or PCuS-MG, n = 3. (b) *Ex vivo* fluorescence imaging of major organs and tumor tissues collected from mice at 48 h post-injection of PCuS or PCuS-MG. (c) Quantification of the fluorescence signal intensity in organs and tumors. (d) Schematic illustration of the *in vivo* treatment schedule and experimental groups. G1 (Control), G2 (CuS), G3 (PCuS), G4 (PCuS-MG), and G5 (PCuS-MG + Laser), n = 5. (e) Tumor volume of 4T1 tumor-bearing mice in different treatment groups. (f) Tumor weight in different treatment groups at day 15 post-treatment. (g) Survival rates of mice in different treatment groups. (h) Body weight changes of mice in different treatment groups. (i) H&E staining of major organs from mice. Scale bar = 250 μm. (j-m) Serum levels of liver function markers (ALT, AST) and kidney function markers (BUN, Crea) in mice from different treatment groups, n = 5.

To evaluate the *in vivo* biodistribution and tumor-targeting capability of PCuS-MG, we intravenously injected 4T1 tumor-bearing mice with either PCuS or PCuS-MG and performed *in vivo* fluorescence imaging at different time points. As shown in Fig. 4a, PCuS-MG exhibited a time-dependent accumulation in the tumor site, with strong fluorescence signals detected at the tumor region as early as 24 h and peaking at 48 h. In contrast, PCuS showed weaker tumor accumulation throughout the observation period. *Ex vivo* fluorescence imaging of major organs and tumors at 48 h post-injection confirmed that PCuS-MG displayed significantly higher fluorescence intensity in the tumor tissue compared to PCuS, while the fluorescence signals in normal organs were relatively low (Fig. 4b). Quantitative analysis further demonstrated that PCuS-MG achieved significantly higher tumor enrichment and lower liver fluorescence signals than PCuS (Fig. 4c), indicating improved targeting and reduced off-target distribution. The hypoxia tropism of PCuS-MG was detected by immunofluorescence staining. In the hypoxic areas of the tumor, the distribution of PCuS-MG shows a significant co-localization with the expression of HIF-1α (Fig. S13). In contrast, in the non-hypoxic areas of the tumor, the distribution of PCuS-MG is more localized, which indicates that PCuS-MG can selectively accumulating in the hypoxic regions of the tumor.

### 2.5 *In vivo* antitumor efficacy and biosafety of PCuS-MG

Next, we evaluated the *in vivo* antitumor efficacy of PCuS-MG using a 4T1 subcutaneous tumor model. The treatment schedule and experimental groups are illustrated in Fig. 4d: mice were randomly divided into five groups (G1: Control, G2: CuS, G3: PCuS, G4: PCuS-MG, G5: PCuS-MG + Laser) and received intravenous injections of the corresponding formulations followed by 1064 nm NIR laser irradiation. Tumor volume was measured every 2 days, and the results showed that tumor growth in the PCuS-MG + Laser group (G5) was significantly inhibited compared to all other groups (Fig. 4e, Fig. S14). At day 15 post-treatment, the tumor weight in the G5 group was markedly lower than that in the control (G1), CuS (G2), PCuS (G3), and PCuS-MG (G4) groups (Fig. 4f). Mice in the G5 group had a significantly prolonged median survival time compared to other groups (Fig. 4g). Notably, no significant changes in body weight were observed in any of the treatment groups throughout the experiment (Fig. 4h), indicating that the treatment was well-tolerated with minimal systemic toxicity.

To further assess the *in vivo* biosafety of PCuS-MG, we performed histological analysis of major organs using hematoxylin and eosin (H&E) staining at 7 days post-treatment. As shown in Fig. 4i, no obvious pathological abnormalities or tissue damage were observed in the PCuS-MG (G4) or PCuS-MG + Laser (G5) groups, and the tissue morphology was comparable to that of the control group (G1). Additionally, we measured serum levels of liver function markers (alanine aminotransferase, ALT; aspartate aminotransferase, AST) and kidney function markers (blood urea nitrogen, BUN; creatinine, Crea) to evaluate potential organ toxicity. The results showed that there were no significant differences in the levels of these biochemical markers between the treatment groups and the control group (Fig. 4j–m). The serum levels of IFN-γ, TNF-α, and IL-6 showed no significant differences between groups, indicating no excessive systemic inflammation (Fig. S15a–c). H&E staining of spleen sections showed that the overall architecture of the spleen remained intact in all groups. There was no evidence of excessive immune activation, such as abnormal white pulp hyperplasia or massive inflammatory cell infiltration(Fig. S15d). These results confirm that PCuS-MG has excellent *in vivo* biosafety and does not cause significant organs damage.

### 2.6 *In vivo* antitumor effects of PCuS-MG in bilateral tumor and lung metastasis models

**Fig. 5:**
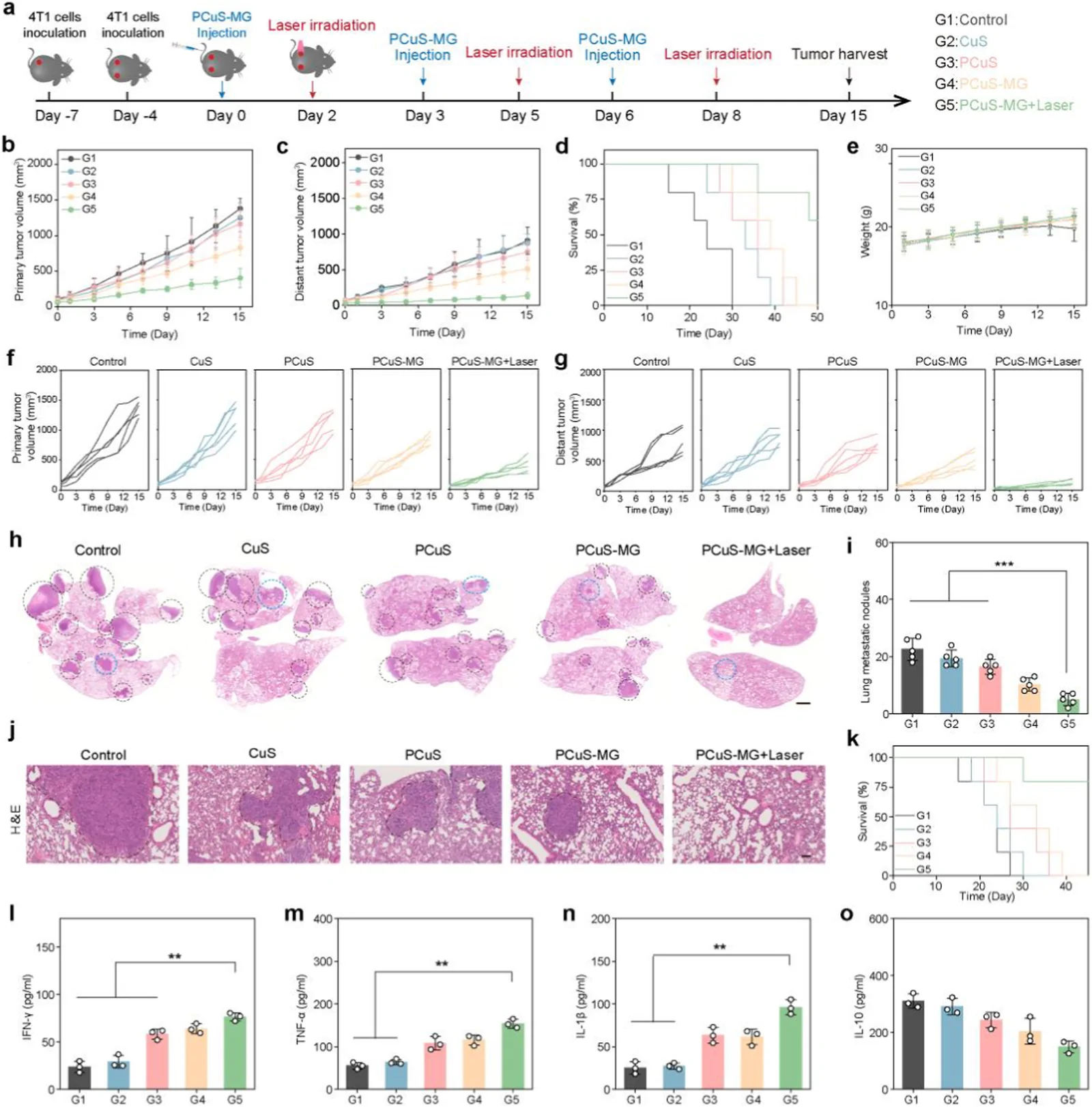
**PCuS-MG + Laser inhibits tumor growth and lung metastasis.** (a) Schematic illustration of the treatment schedule and experimental groups in the bilateral subcutaneous 4T1 tumor model, n = 5. (b) Primary tumor volume of mice in different treatment groups. (c) Distant tumor volume of mice in different treatment groups. (d) Survival rates of mice in the different treatment groups. (e) Body weight changes of mice in different treatment groups. (f, g) Individual primary (f) and distant (g) tumor growth curves of mice in different treatment groups. (h) Representative H&E staining images of lung tissues from mice in different treatment groups. Black circles indicate metastatic nodules, and blue circles mark the specific regions selected for the high-magnification views in panel (j). Scale bar = 500 μm. (i) Quantitative analysis of the number of lung metastatic nodules in different treatment groups, n = 5. (j) High-magnification H&E-stained images of lung tissue. Scale bar = 100 μm. (k) Survival rates of mice in the 4T1 lung metastasis model. (l-o) ELISA analysis of pro-inflammatory cytokines (IFN-γ, TNF-α, IL-1β) and anti-inflammatory cytokine (IL-10) levels in blood from mice in different treatment groups, n = 3. (For interpretation of the references to colour in this figure legend, the reader is referred to the Web version of this article.)

To evaluate the systemic antitumor effect of PCuS-MG, we established a bilateral subcutaneous 4T1 tumor model, followed by treatment according to the schedule shown in Fig. 5a. Mice were randomly divided into five groups: G1 (Control), G2 (CuS), G3 (PCuS), G4 (PCuS-MG), and G5 (PCuS-MG + Laser). As shown in Fig. 5b–c, the CuS and PCuS groups showed no significant difference in the growth of primary or distant tumors compared to the Control group, suggesting that a lack of targeting limits sufficient drug accumulation at the tumor site. In contrast, PCuS-MG treatment alone inhibited the growth of both primary and distant tumors, confirming that bacterial delivery enhances the anti-tumor activity of the therapeutic payload (Fig. 5f and g). The most potent effect was observed in the PCuS-MG + Laser group, which achieved inhibition rates of 84.3% for primary tumors and 73.9% for distant tumors. Consequently, 60% of the mice in this group survived for over 60 days, a significant improvement over all other groups (Fig. 5d). Further, no significant body weight loss was observed in any group (Fig. 5e). In addition, we also evaluated the *in vivo* anti-tumor efficacy of pirfenidone alone and *E. coli* MG1655 alone (Fig. S16). Compared with the control group, both single-agent treatments only achieved moderate and partial tumor growth inhibition. The *E. coli* MG1655 alone group showed slightly stronger inhibition than pirfenidone group, but tumors still grew progressively, demonstrating that neither pirfenidone nor *E. coli* MG1655 alone is sufficient to produce robust anti-tumor efficacy.

Next, we assessed the antimetastatic efficacy of PCuS-MG in a 4T1 lung metastasis model (Fig. S17). H&E staining of lung tissues revealed that the number of metastatic nodules was markedly reduced in the G5 group compared to other groups (Fig. 5h–j). Quantitative analysis confirmed that the number of lung metastatic nodules in G5 was significantly lower than in G1, G2, G3, and G4 (Fig. 5i). Survival analysis demonstrated that mice in G5 had a significantly prolonged survival time of mice compared to other groups (Fig. 5k), further validating the antimetastatic efficacy of PCuS-MG + Laser. To investigate the systemic immunostimulatory effects of PCuS-MG, we measured the cytokines levels in blood. ELISA results showed that the levels of pro-inflammatory cytokines, including IFN-γ, TNF-α, and iIL-1β, were significantly higher in the G5 group (Fig. 5l–n) to the control group. In contrast, the level of the anti-inflammatory cytokine IL-10 was significantly lower in G5 compared to other groups (Fig. 5o). These results indicate that PCuS-MG + Laser effectively activate a systemic anti-tumor immune response, contributing to the inhibition of distant tumors growth and lung metastasis.

### 2.7 Evaluation of antitumor immune mechanisms of PCuS-MG

**Fig. 6:**
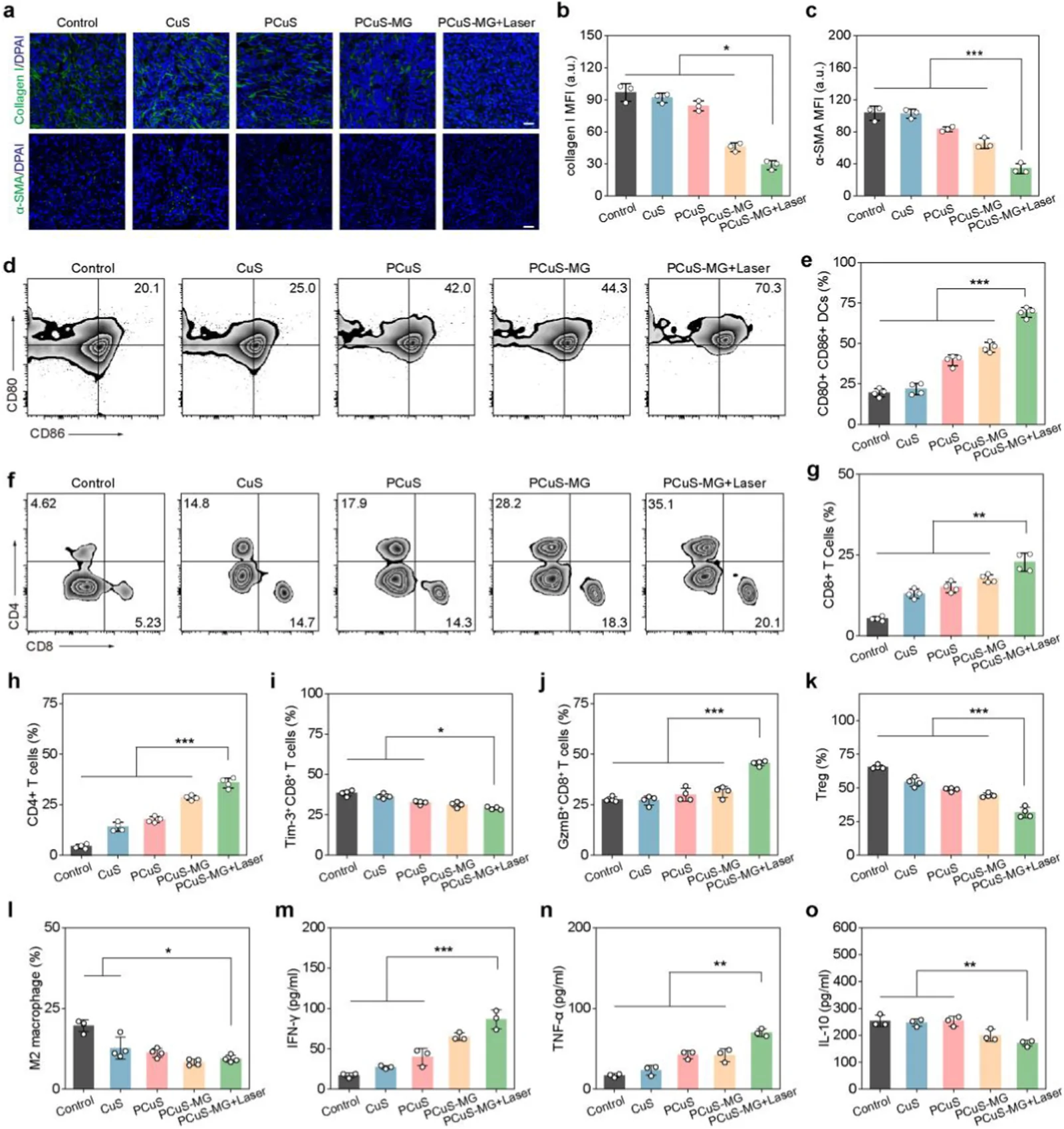
**Analysis of the tumor immune microenvironment and CAFs modulation.** (a) Immunofluorescence staining of collagen I and α-SMA in tumor tissues from mice in different treatment groups. Scale bar = 100 μm. (b, c) Quantitative analysis of the MFI of collagen I and α-SMA in tumor tissues, n = 3. (d-e) Flow cytometry plots and quantification of mature DCs in tumors, n = 4. (f) Flow cytometry plots of CD4^+^ and CD8^+^ T cells in tumor tissues from mice in different treatment groups, n = 4. (g, h) Quantitative analysis of the percentage of CD8^+^ T cells (g) and CD4^+^ T cells (h) in tumor tissues. (i, j) Quantitative analysis of the percentage of Tim-3^+^ CD8^+^ T cells (i) and Granzyme B^+^ CD8^+^ T cells (j) in tumor tissues. (k) Quantification of the proportion of Tregs in tumors. (l) Quantification of M2-type macrophages in tumors. (m-o) ELISA analysis of the levels of IFN-γ, TNF-α and IL-10 in tumor tissues from mice in different treatment groups, n = 3.

To clarify the immunomodulatory mechanisms responsible for the potent anti-tumor effect of PCuS-MG, we conducted a systematic analysis of the immune microenvironment in treated 4T1 tumor tissues. Immunofluorescence staining of tumor sections provided further evidence of CAFs modulation. The PCuS-MG + Laser treatment significantly elevated DLAT expression, confirming robust cuproptosis induction in tumor tissues (Fig. S18). Importantly, ATP7A expression was significantly downregulated in the PCuS-MG + Laser group (Fig. S19a), while the copper ion concentration in tumor tissues was significantly elevated in this group (Fig. S19b). Furthermore, we found that the PCuS-MG + Laser treatment significantly reduced the deposition of the CAFs activation marker α-SMA and type I collagen compared to the Control group (Fig. 6a–c). This confirms that the treatment successfully suppressed CAFs activation, reduced ECM deposition, and decreased tissue stiffness, facilitating enhanced immune cell infiltration. Flow cytometry data indicated that PCuS-MG combined with laser irradiation significantly enhanced the maturation of DCs in tumor-draining lymph nodes, resulting in a 2.85-fold increase compared to the Control group (Fig. 6d–e, Fig. S20). This activation of the innate immune system subsequently amplified adaptive immune responses. Analysis of tumor-infiltrating T cell populations revealed that the PCuS-MG + Laser group had significantly higher proportions of both CD8^+^ and CD4^+^ T cells within tumors compared to other groups (Fig. 6f–h, Figure S21-22). Furthermore, this treatment induced the most robust T cell activation, elevating the percentage of functional CD8^+^ T cells from 27.6 ± 1.24% in the Control group to 45.53 ± 1.27% (Fig. 6j). A substantial decrease in exhausted CD8^+^ T cells was also observed, indicating a reversal of T cell dysfunction and enhanced cytotoxic capacity (Fig. 6i). Conversely, the populations of immunosuppressive cells, including regulatory T cells (Tregs) and M2-type macrophages, were markedly reduced in the PCuS-MG + Laser group (Fig. 6k–l, Fig. S23). This shift in the immune cell landscape is likely due to pirfenidone released from PCuS-MG, which remodels the extracellular matrix by modulating CAFs and thereby alleviates immunosuppression in the TME. These cellular changes were accompanied by significant alterations in cytokine levels. Tumors from the PCuS-MG + Laser group exhibited a pronounced increase in the pro-inflammatory cytokines TNF-α and IFN-γ, alongside a marked decrease in the immunosuppressive cytokine IL-10 (Fig. 6m–o). These findings collectively confirm that PCuS-MG effectively activates DCs-mediated anti-tumor immunity.

In summary, PCuS-MG therapy enhances cuproptosis and anti-tumor immunotherapy through following mechanism: (i) Under 1064 nm laser irradiation, PCuS-MG enables controlled release of copper ions and pirfenidone within tumors, inducing cuproptosis, ferroptosis, and ICD; (ii) The released pirfenidone inhibits CAFs activation and remodels the extracellular matrix, thereby reducing tumor invasiveness and resistance to cuproptosis; (iii) Remodeling of the tumor microenvironment creates a favorable metabolic milieu for the maturation of DCs, ultimately triggering a robust systemic antitumor immune response.

## 3 Conclusion

To address the scientific challenges of treatment failure caused by cuproptosis resistance and the immunosuppressive tumor microenvironment, this study successfully developed a "Trojan bacteria" system, PCuS-MG. This platform is designed to synergistically enhance cuproptosis therapy and immunotherapy through the targeted modulation of CAFs and metabolic reprogramming. Leveraging the hypoxic tropism of *E. coli* MG1655, PCuS-MG achieves targeted delivery to tumors. Upon 1064 nm laser irradiation, the photothermal effect lyses the bacterial carriers, enabling the controlled release of copper ions and pirfenidone directly at the tumor site. The released copper ions synergistically induce cuproptosis and ferroptosis by promoting the aggregation of DLAT and downregulating GPX4, concurrently generating substantial cytotoxic ROS. More importantly, we found that CAFs serve as the primary driver of cuproptosis resistance, rather than a secondary bystander factor in the tumor microenvironment. In vitro co-culture results showed that direct PFD treatment exerted almost no effect on ATP7A expression in 4T1 tumor cells. Nevertheless, co-culturing tumor cells with CAFs significantly upregulated ATP7A, which promoted copper efflux and induced cuproptosis resistance. This cuproptosis resistance could be reversed after PFD-mediated CAFs reprogramming, confirming that CAFs are required to trigger cuproptosis resistance. In vivo data demonstrated that the PCuS-MG + Laser group achieved obvious CAFs reprogramming within tumor tissue, accompanied by reduced ATP7A expression, elevated intratumoral copper content, and increased expression of the cuproptosis marker. Furthermore, effective CAFs reprogramming also remodels the ECM and reduces collagen deposition, ultimately leading to enhanced DCs infiltration and functional activation of CD8^+^ cytotoxic T cells.

In summary, PCuS-MG significantly amplifies cuproptosis and the subsequent anti-tumor immune response through the synergistic inhibition of CAFs and regulation of glucose metabolism. This approach not only potently inhibits primary tumor growth but also triggers a systemic abscopal effect, suppressing the growth of distant tumors and lung metastases. These findings establish PCuS-MG as a promising therapeutic platform that combines metal ion-induced cell death with targeted tumor microenvironment modulation, offering a novel strategy to overcome current bottlenecks in cancer therapy. Different from previous studies that merely applied bacteria, CuS nanoparticles or pirfenidone alone, our work is not a simple combination of existing materials and strategies. Instead, we clarify an original regulatory axis that CAF activation intrinsically drives tumor cuproptosis resistance via TGF-β signaling and glucose metabolism reprogramming. *In vitro* and *in vivo* evidence further corroborates that CAFs serve as the primary driver of cuproptosis resistance in the tumor microenvironment. Targeted CAF reprogramming therefore represents a pivotal strategy to reverse cuproptosis resistance and amplify immunotherapeutic efficacy. Future work should focus on elucidating the multidimensional interactions between metal ion-induced death, CAFs, metabolic reprogramming, and immune cells. Clarifying the synergistic regulatory network of pirfenidone and copper ions, alongside optimizing the nanoplatform's biosafety and delivery efficiency, will provide a stronger foundation for the clinical translation of cuproptosis-based therapies.

## CRediT authorship contribution statement

**Yuhao Chen:** Writing – original draft, Methodology, Conceptualization. **Xiaorui Geng:** Writing – original draft, Methodology. **Ning Su:** Methodology, Investigation. **Phei Er Saw:** Writing – review & editing, Formal analysis. **Yun Li:** Investigation. **Bin Liu:** Validation. **Yingjia Li:** Project administration. **Zhen Yuan:** Writing – review & editing, Resources, Project administration.

## Declaration of competing interests

The authors declare that they have no known competing financial interests or personal relationships that could have appeared to influence the work reported in this paper.

## Data Availability

Data will be made available on request.
