# Supplementary material for: Engineered bacteria reverses tumor cuproptosis resistance via cancer-associated fibroblast reprogramming for enhanced immunotherapy
Source: Mater Today Bio. 2026 Aug 26;40:103603. doi: 10.1016/j.mtbio.2026.103603 (PMC13545382; doi:10.1016/j.mtbio.2026.103603)
Supplement: Supplementary file 1 [file mmc1.docx]

Supporting Information for

**Engineered Bacteria Reverses Tumor Cuproptosis Resistance via Cancer-Associated Fibroblast Reprogramming for Enhanced Immunotherapy**

Yuhao Chen^1,2#^, Xiaorui Geng^1,2#^ , Ning Su^1,3#^, Phei Er Saw^4^, Yun Li^5^, Bin Liu^6^, Yingjia Li^3^, Zhen Yuan^1,2*^

^1^Cancer Center, Faculty of Health Sciences, University of Macau, Macau Special Administrative Region of China

^2^Centre for Cognitive and Brain Sciences, University of Macau, Macau Special

Administrative Region of China

^3^Department of Medicine Ultrasonics, Nanfang Hospital, Southern Medical University, Guangzhou, China

^4^Medical Research Center, Sun Yat-sen Memorial Hospital, Sun Yat-sen University, Guangzhou, China

^5^ Guangdong Yunzhao Medical Technology Co., LTD, Guangzhou, China

^6^ Department of Emergency, Zhujiang Hospital, Southern Medical University, Guangzhou,

Guangdong Province, China

*Corresponding author: [zhenyuan@um.edu.mo](mailto:zhenyuan@um.edu.mo); # represents equal contribution to this work

**Method**

**Construction of In Vitro Co-Culture Model of CAFs and Tumor Cells**

Normal 3T3 fibroblasts were inoculated in the upper layer of Transwell insert (1×10^4^ per well). The 3T3 fibroblasts were cultured with recombinant transforming growth factor-β1 (TGF-β1, 10 ng/ml) by 48h to induce them transdifferentiate into CAFs. 4T1 tumor cells were inoculated in 24-well plates (5×10^4^ per well), thereby achieving co-culture of CAFs and 4T1 cells. This setup established a defined CAF-to-4T1 cell co-culture ratio of 1:5 (1 × 10⁴ CAFs in the upper insert and 5 × 10⁴ 4T1 cells in the lower well). The co-culture system was then incubated at 37 °C in a humidified atmosphere with 5% CO₂ for an additional 48 h. CAFs were fixed with 4% paraformaldehyde and received blocking treatment for 30 minutes. Anti-α-SMA or anti-Collagen I antibody was added and incubated at 4 °C for 12 hours. Then, fluorescently labeled secondary antibody was added for 2 hours of incubation, and cell nuclei were stained with DAPI. The expression of α-SMA or Collagen I in treated CAFs was observed by confocal fluorescence microscope. The culture supernatant of CAFs was collected, and the concentrations of TGF-β and HGF were detected using ELISA kits. 4T1 cells were digested and collected, and the expression of ATP7A in 4T1 cells was detected by flow cytometry.

**Evaluation of Glucose Metabolism**

CAFs were inoculated in 24-well plates and cultured overnight at 37 °C. The glucose level in the tumor cell culture supernatant was detected using Glucose Colorimetric Assay Kit, and the glucose uptake was calculated. The lactate production level in the tumor cell culture supernatant was detected using L-Lactate Assay Kit. The extracellular acidification rate (ECAR) and oxygen consumption rate (OCR) of tumor cells were evaluated using a Seahorse XFe96 Analyzer (Agilent) following the instrument’s standard experimental protocol. 4T1 cells were inoculated in specialized 96-well plates and received corresponding therapeutic treatments. For ECAR assessment, the cell medium was replaced with specialized XF DMEM medium containing glutamine. Glucose (100 mM), oligomycin (10 μM), and 2-DG (500 mM) were added at preset time points, and various parameters of ECAR were quantitatively analyzed using Seahorse Wave software. For OCR assessment, the cell medium was replaced with specialized XF DMEM medium containing glucose, glutamine, and pyruvate. Oligomycin (15 μM), FCCP (20 μM), and rotenone/antimycin A (5 μM) were added at preset time points, and various parameters of OCR were quantitatively analyzed using Seahorse Wave software.

**Preparation of PCuS-MG**

First, PCuS nanoparticles were prepared: A BSA solution with a concentration of 40 mg/mL was prepared. Copper chloride solution (0.2 M) was added to the BSA solution, followed by rapid stirring. Subsequently, sodium sulfide solution (0.2 M) was added to the mixture, and the solution was stirred vigorously for 50 minutes. The resulting solution was centrifuged for 20 minutes to collect CuS nanoparticles. The pirfenidone solution (10 mg/mL) was mixed with the CuS nanoparticle solution (1 mg/mL) and stirred at room temperature for 6 hours to allow pirfenidone to enter the hydrophobic pockets of BSA through hydrophobic interactions. The reaction solution was dialyzed (MWCO 3500 Da) to remove free drugs. The synthesized drug-loaded copper sulfide nanoparticles (200 μL, 10 mg/mL) were reacted with glucose polymer (*poly [4-O-(α-D-glucopyranosyl)-D-glucopyranose]*) (100 μL, 10 mg/mL) at 37°C for 8 hours. Then, 0.05 mg of NaBH₄ was added, and the mixture was stirred under ice bathing for 2 hours. The PCuS nanoparticles were collected by centrifugation for subsequent experiments. To construct PCuS-MG, monoclonal *E. coli* MG1655 was selected and inoculated in LB liquid medium, followed by cultivation at 37°C. When the OD_600_ value of the bacteria reached 0.5, the bacteria were collected by centrifugation. E. coli MG1655 at a concentration of 1.0×10⁷ CFU was incubated with PCuS (15 mg/mL) at 200 rpm for 2 hours. PBS was used for washing to remove excess PCuS, and the constructed PCuS-MG was collected by centrifugation at 5000 rpm for 10 minutes.

**Characterization of PCuS-MG**

The hydrodynamic diameter and surface charge of PCuS nanoparticles were detected by dynamic light scattering (DLS). The UV absorption spectrum of PCuS was measured using a UV-Vis spectrophotometer. Fluorescence microscope and flow cytometry was employed to verify whether PCuS internalized with *E. coli* MG1655. Additionally, the morphology of PCuS-MG was observed via scanning electron microscopy (SEM).

***In Vitro* Drug Release**

PCuS-MG solution was added to a 96-well plate, and the temperature change of the solution under 1064 nm laser irradiation (1 W/cm²) was monitored using an infrared thermal imager. The viability of bacteria after laser irradiation was detected by the CCK-8 assay. Laser-irradiated PCuS-MG was placed in dialysis bags (molecular weight: 14000 Da), which were then immersed in PBS buffer containing sodium dodecyl sulfate (SDS) and incubated at 37 °C with gentle stirring. At different time points, 1 mL of PBS solution was collected to be measured the absorbance at 310 nm using a UV-Vis spectrophotometer to evaluate the release of pirfenidone.

**Detection of GSH Consumption and Cu⁺ Generation**

PCuS-MG solutions of different concentrations were added to a 96-well plate and exposed to 1064 nm laser irradiation (1 W/cm², 3 min). GSH solution (10 mM) was added to the wells and incubated for 30 min, followed by the addition of DTNB solution (3 mM) for a 30-min light-protected reaction. A microplate reader was used to measure the absorbance at 412 nm to calculate the GSH consumption rate. The neocuproine solution (5 mM) was added to the wells. After a 15-min reaction, the absorbance at 450 nm was detected using a microplate reader to quantify Cu⁺ generation.

**In Vitro Cellular Uptake Assay**

4T1 cells were inoculated in 24-well plates at a density of 5×10⁴ cells per well. The cells were co-incubated with Cy5.5-labeled PCuS or PCuS-MG. After incubation, the cells were fixed with 4% paraformaldehyde, and cell nuclei were stained with DAPI. The cellular uptake of PCuS-MG was observed by fluorescence microscopy and quantitatively analyzed by flow cytometry.

**In Vitro Cytotoxicity Assay**

4T1 cells were inoculated in 96-well plates at a density of 10,000 cells per well and received corresponding therapeutic treatments. Calcein and PI solutions were added to the wells, followed by incubation at 37 °C for 30 min. Live/dead cells staining were observed by fluorescence microscopy. Alternatively, medium containing CCK-8 reagent was added to the wells, and a microplate reader was used to measure the absorbance at 450 nm for quantitative analysis of 4T1 cell viability.

**Detection of Intracellular ROS Levels**

4T1 cells were inoculated in 96-well plates and received corresponding therapeutic treatments. Medium containing DCFH-DA probe was added to the wells, followed by incubation in the dark for 30 minutes. After washing with PBS, the green fluorescent signal in 4T1 cells was analyzed by fluorescence microscopy and flow cytometry to evaluate the intracellular ROS level.

**Detection of Mitochondrial Membrane Potential**

4T1 cells were inoculated in 96-well plates and received corresponding therapeutic treatments. JC-1 staining solution was added to the wells and incubated in the dark for 30 minutes. After washing, the fluorescent signals of JC-1 monomers and JC-1 aggregates were observed by fluorescence microscopy to assess changes in mitochondrial membrane potential of 4T1 cells.

**Detection of Intracellular ATP Content**

4T1 cells were inoculated in 24-well plates and received corresponding therapeutic treatments. The lysis buffer was added to lyse the cells, and the assay was performed according to the instructions of the ATP detection kit to quantitatively analyze the intracellular ATP content in 4T1 cells after treatment.

**Immunofluorescence Staining of Tumor Cells**

4T1 cells were inoculated in 24-well plates and cultured overnight at 37 °C. The cells were received corresponding therapeutic treatments and then fixed with 4% paraformaldehyde. 4T1 cells were treated with blocking solution for 30 minutes. After washing with PBS, anti-DLAT or anti-GXP4 antibody was added and incubated at 4 °C for 12 hours. Subsequently, fluorescently labeled secondary antibody was added for 2 hours of incubation. After washing with PBS, nuclei were stained with DAPI for 5 minutes. The expression of DLAT or GXP4 in treated 4T1 cells was observed by confocal fluorescence microscope.

**Detection of Intracellular Lipid Peroxidation**

4T1 cells were inoculated in 24-well plates and cultured overnight at 37 °C. The cells were received corresponding therapeutic treatments. Cells were digested with trypsin, collected, and incubated with BODIPY 581/591 C11 staining solution for 30 minutes. After washing with PBS, flow cytometry was used to analyze the fluorescence intensity of the cells, and the lipid peroxidation level was calculated.

**Detection of DAMPs Release**

4T1 cells were inoculated in 24-well plates and cultured overnight at 37 °C. The cells were received corresponding therapeutic treatments and then fixed with 4% paraformaldehyde. 4T1 cells were treated with blocking solution for 30 minutes. After washing with PBS, anti-CRT antibody was added and incubated at 4 °C for 12 hours. Subsequently, fluorescently labeled secondary antibody was added for 2 hours of incubation. After washing with PBS, nuclei were stained with DAPI for 5 minutes. The expression of CRT in treated 4T1 cells was observed by confocal fluorescence microscope. The culture supernatant of 4T1 cells was collected, and the concentration of HMGB1 in the medium was detected using ELISA kit.

**Evaluation of DCs Maturation**

4T1 cells were inoculated in 24-well plates and cultured overnight at 37 °C. The cells were received corresponding therapeutic treatments, and the culture supernatant of 4T1 cells was collected 6 hours later. DCs were inoculated in 24-well plates, and the 4T1 cell culture supernatant was added for co-incubation with DCs for 24 hours. DCs were digested with trypsin and collected, then incubated with CD80 and CD86 flow cytometry antibodies for 30 minutes. The expression of CD80 and CD86 on DCs was detected by flow cytometry. The culture supernatant of DCs was collected, and the concentrations of IL-6, TNF-α, IL-12p70, and IL-10 in the medium were detected using ELISA kits.

**In Vivo Biodistribution of PCuS-MG**

BALB/c mice (female, 6–8 weeks old) were obtained from the Animal Research Core, Faculty of Health Sciences, University of Macau. 4T1 cells (1×10⁶ per mouse) were subcutaneously injected into the right flank of BALB/c mice to establish the subcutaneous tumor-bearing model. When the tumor volume reached approximately 200 mm³, the tumor-bearing mice were randomly divided into 2 groups (n=3). The mice were intravenously injected with DiR-labeled PCuS or PCuS-MG (2 mg/kg PCuS). In vivo fluorescence imaging was performed using a small animal in vivo imaging system at different time points after drug injection. Mice were euthanized 48 hours after injection, and organs and tumors were collected for ex vivo imaging and quantitative analysis of fluorescence signal intensity.

**Antitumor Efficacy and Immunotherapeutic Study of PCuS-MG**

The primary tumor mouse model was established by subcutaneously injecting 1×10⁶ 4T1 cells into the right flank of Balb/c mice. Three days later, 1×10⁶ 4T1 cells were subcutaneously injected into the contralateral flank to establish the distant tumor model. When the volume of primary tumors reached 50–100 mm³, the tumor-bearing mice were randomly divided into five groups (n=5): (i) Control group, (ii) CuS group, (iii) PCuS group, (iv) PCuS-MG group, and (v) PCuS-MG + Laser group. Mice received treatments on Days 7, 10, and 13, respectively. Mice in the Control, CuS, and PCuS groups were intravenously injected with PBS, CuS, or PCuS (2 mg/kg). Mice in the PCuS-MG and PCuS-MG + Laser groups were intravenously injected with PCuS-MG (2 mg/kg PCuS). The primary tumors of mice in the PCuS-MG + Laser group were received laser irradiation (1064 nm, 1 W/cm², 3 min) after 48 hours of injection. Tumor volume and body weight of mice were recorded every 2 days. When the tumor volume exceeded 2000 mm³, mice were euthanized by carbon dioxide asphyxiation, and the survival time was recorded. In addition, to explore the effect of treatment on the tumor immune microenvironment, 7 days after the treatment, tumor-draining lymph nodes and tumors were collected from mice. Tissues were carefully minced and digested with collagenase and DNase to prepare single-cell suspensions. The single-cell suspensions were used for flow cytometric analysis of dendritic cells, T cells, and macrophages, as well as ELISA detection of cytokines. Tumor tissues were prepared into paraffin sections, and the expression of α-SMA and Collagen I was observed by immunofluorescence staining.

**Statistical Analysis**

Statistical analyses were performed by SPSS 26.0 software. One-way analysis of variance (ANOVA) with Tukey post hoc tests was used to evaluate comparisons among multiple groups of experimental data. Independent samples t-test was applied for comparisons between two groups of data. All experimental data were presented as mean ± standard deviation (SD). Statistical significance was defined as **P* ≤ 0.05, ***P* < 0.01, and ****P* < 0.001.

Figure S1. The mean fluorescence intensity (MFI) of immunofluorescence staining of α-SMA and Collagen I in CAFs after different treatments.

Figure S2. mRNA expression of glycolytic genes HK2 and LDHA in CAFs after different treatments.

Figure S3. OCR quantitative analysis of basal respiration, ATP production, and maximal respiration in CAFs after different treatments.

Figure S4. ECAR quantitative analysis of glycolytic rate, glycolytic capacity, and glycolytic reserve in CAFs after different treatments.

Figure S5. Quantification of ATP7A expression in tumor cells after different treatments.

Figure S6. ATP7A expression in 4T1 tumor cells under different treatments. (a) Representative flow cytometry showing ATP7A expression in 4T1 cells treated with Cu²⁺, PFD, and co-cultured with CAFs. (b) Quantitative analysis of ATP7A expression levels.

Figure S7. Characterization of PCuS-MG. (a) Zeta potential of CuS, PCuS, and PCuS-MG. (b) Standard curve for PFD concentration determination, used to calculate the drug encapsulation efficiency of PCuS-MG.

 Figure S8. Viability of PCuS-MG after exposure to 1064 nm laser with different time.

Figure S9. Stability of PCuS nanoparticles over 5 days.

 Figure S10. Flow cytometry of tumor cells uptake of PCuS or PCuS-MG.

Figure S11. (a-b) DCFH-DA staining of tumor cells after different treatments. (c) GSH/GSSG of tumor cells after different treatments.

Figure S12. Flow cytometry of lipid peroxidation in tumor cells after different treatments.

Figure S13. Immunofluorescence co-localization of PCuS-MG with the hypoxia marker HIF-1α in tumor tissues. Top row: Low-magnification immunofluorescence images of tumor tissue. Two white boxes in the merged figure mark the hypoxic and non-hypoxic regions, which correspond to the high-magnification views. Middle row: High-magnification images of the hypoxic tumor area. Bottom row: High-magnification images of the non-hypoxic tumor area. Scale bars: 200 μm (low magnification), 50 μm (high magnification).

Figure S14. Individual tumor volume growth curves of 4T1 tumor-bearing mice in different treatment groups (n=5).

Figure S15. Systemic safety evaluation of PCuS-MG therapy. (a–c) Serum levels of pro-inflammatory cytokines IFN-γ, TNF-α, and IL-6. (d) H&E staining of spleen sections. Scale bar: 50 μm.

Figure S16. In vivo anti-tumor efficacy of pirfenidone and *E. coli* MG1655 treatments. (a, b) Growth curves of primary and distant tumors in Control, Pirfenidone, and E. coli MG1655 groups, n=5. (c) Survival curves of tumor-bearing mice in each group. (d) Body weight changes of mice during treatment. (e, f) Individual growth curves of primary and distant tumors for each mouse in the three groups.

Figure S17. Schematic of the treatment protocol for the 4T1 lung metastasis tumor-bearing mouse models.

Figure S18. Immunofluorescence staining of the cuproptosis marker DLAT in tumor tissues. Scale bar: 25 μm.

Figure S19. ATP7A expression and copper content in tumor tissues after different treatments.(a) Representative immunofluorescence images of ATP7A (green) and DAPI (blue) in tumor sections. Scale bar: 50 μm. (b) Quantitative analysis of copper ion concentration in tumor tissues.

Figure S20. Representative flow cytometry gating strategy for analyzing DC maturation.

Figure S21. Immunofluorescence staining of T cells in tumor tissues from different treatment groups. T cell infiltration labeled with CD4 (green) and CD8 (red).

Figure S22. Representative flow cytometry gating strategy for analyzing T cell infiltration.

Figure S23. Quantification of M1 phenotype macrophages in tumors.
